# Peer Review of “A Machine Learning Explanation of the Pathogen-Immune Relationship of SARS-CoV-2 (COVID-19), and a Model to Predict Immunity and Therapeutic Opportunity: A Comparative Effectiveness Research Study”

**DOI:** 10.2196/24765

**Published:** 2020-10-19

**Authors:** Eric Abbott

**Affiliations:** 1 Medical Health Informatics Graduate Program Northwestern University Chicago, IL United States

**Keywords:** infectious disease, SARS-CoV-2, COVID-19, public health, immunity: vaccinations, therapeutics, stem-cell growth factor-beta

*This is a peer review submitted for the paper “A Machine Learning Explanation of the Pathogen-Immune Relationship of SARS-CoV-2 (COVID-19), and a Model to Predict Immunity and Therapeutic Opportunity: A Comparative Effectiveness Research Study.”*

## General Comments

This paper is excellent and very timely given its importance as it relates to supporting forthcoming mass vaccinations to address COVID-19 and potential prioritization of such vaccinations based on the study findings.

## Specific Comments

### Major Comments

1. None

2. None

3. None

### Minor Comments

4. Consider consistency of using COVID-19 vs SARS-CoV-2 (abstract vs text body).

